# Nature should be the model for microbial sciences

**DOI:** 10.1128/jb.00228-24

**Published:** 2024-08-19

**Authors:** Brett J. Baker, Emily Hyde, Pedro Leão

**Affiliations:** 1 Department of Marine Science, University of Texas at Austin, Marine Science Institute, Port Aransas, Texas, USA; 2 Department of Integrative Biology, University of Texas at Austin, Austin, Texas, USA; 3 Department of Microbiology—RIBES, Radboud University, Nijmegen, the Netherlands; Geisel School of Medicine at Dartmouth, Hanover, New Hampshire, USA

**Keywords:** Archaea, Asgardarchaeota, SAR11, Hadesarchaeia, Bathyarchaeia, *Pelagibacter*, Heimdallarchaeia, Thaumarchaeota, *Nitrosopumilus*

## Abstract

Until recently, microbiologists have relied on cultures to understand the microbial world. As a result, model organisms have been the focus of research into understanding Bacteria and Archaea at a molecular level. Diversity surveys and metagenomic sequencing have revealed that these model species are often present in low abundance in the environment; instead, there are microbial taxa that are cosmopolitan in nature. Due to the numerical dominance of these microorganisms and the size of their habitats, these lineages comprise mind-boggling population sizes upward of 10^28^ cells on the planet. Many of these dominant groups have cultured representatives and have been shown to be involved in mediating key processes in nature. Given their importance and the increasing need to understand changes due to climate change, we propose that members of Nitrosophaerota (*Nitrosopumilus maritimus*), SAR11 (*Pelagibacter ubique*), Hadesarchaeia, Bathyarchaeia, and others become models in the future. Abundance should not be the only measure of a good model system; there are other organisms that are well suited to advance our understanding of ecology and evolution. For example, the most well-studied symbiotic bacteria, like *Buchnera*, *Aliivibrio*, and *Rhizobium*, should be models for understanding host-associations. Also, there are organisms that hold new insights into major transitions in the evolution of life on the planet like the Asgard Archaea (Heimdallarchaeia). Innovations in a variety of *in situ* techniques have enabled us to circumvent culturing when studying everything from genetics to physiology. Our deepest understanding of microbiology and its impact on the planet will come from studying these microbes in nature. Laboratory-based studies must be grounded in nature, not the other way around.

## INTRODUCTION

Historically, our understanding of microbes has been based on laboratory cultures. Much of what we know at a mechanistic level is based on “model organisms” which are species that readily grow in laboratory conditions. Model organisms are defined as nonhuman species that serve as subjects in studies aimed at generating knowledge applicable to other species (1). In the realm of microbiology, the species *Escherichia coli* (2) has been the go-to model organism since its discovery in 1884. *E. coli* quickly became the bacterium of choice for laboratory training and experiments. With its rapid growth, adaptability to varying growth conditions, low pathogenicity, and ubiquity in the human microbiome, it was the obvious choice when the molecular biology revolution began in the 1950s. Studies involving *E. coli* played pivotal roles in describing the DNA replication machinery (3), demonstrating the stochastic nature of mutations (4), and serving as the foundation for various genetic engineering technologies, such as molecular cloning (5). It should be noted that we now have thousands of genomes belonging to this species; however, there is considerable genomic variation within these organisms (6). Thus, the concept of *E. coli* as a model is not well defined.

Another bacterium widely employed as a model organism is *Bacillus subtilis*. Like *E. coli*, *B. subtilis* has proven to be amenable to experimental manipulation and boasts a low pathogenicity profile. The prominence of *B. subtilis* as a model stemmed from the need to study Gram-positive bacteria and their distinctive cell wall properties, especially in a medical context. *B. subtilis* also offers unique advantages for the study of cell differentiation, owing to its sporulation capability and ease of biofilm formation (7). In fact, some traits that make them the focus of study are the result of laboratory domestication (8).

In the late 1970s, the emerging technology of nucleic acid sequencing led to the discovery of a new group of methane-producing microorganisms that defied organism classification as eukaryotes or Bacteria (9). This groundbreaking revelation eventually led to the identification of these unique microorganisms now known as Archaea (10, 11). It is worth noting that, even before this taxonomic reclassification, researchers had already collected archaeal cells from acidic ponds (12). Archaea had played pivotal roles in key breakthroughs in cellular biology, such as the discovery of bacteriorhodopsin (13). However, during that era, they were mistakenly grouped together with Bacteria.

Within the domain of Archaea, species from the genus *Sulfolobus* within the TACK superphylum have emerged as the model organisms of choice (14). Moreover, genetic analyses often involve alternatives from the Euryarchaeota phylum, including *Haloferax volcanii*, *Methanosarcina acetivorans*, *Methanococcus maripaludis*, and *Thermococcus gammatolerans*, each with its own unique strengths and limitations (15). Unlike bacterial model organisms, the success of *Sulfolobus* as a model organism can be attributed to its rich array of mobile genetic elements, such as insertion sequences, viruses, and plasmids (16). Understanding and characterizing these genetic elements are pivotal for the development of genetic tools, which are key for exploring the intricacies of archaeal cell biology.

Advances in sequencing technologies and computational approaches in the last 20 years have enabled microbiologists to obtain genomes directly from nature. This has revealed a vast diversity and community structure of dominant lineages previously overlooked and uncultured. Simultaneously, it revealed that the model organisms traditionally employed do not hold a central role in natural ecosystems. Take, for instance, *E. coli*, which is far from being a dominant species in water or soil samples and is often used as a marker for anthropogenic contamination in such environments. This finding has underscored the pressing need for the development of new model organisms that better represent the key players in our ecosystems.

Here, we argue that it is important to focus on the dominant microbes on our planet and how they persist in their natural habitats. Classically, the concept of a model organism relied on an individual species. The concept of a species in microbiology is controversial, and often in nature, populations of species or broad groups (e.g., a phylum) have strong environmental importance. In some cases, the model taxa we describe here have no culture representatives, or there are stable laboratory cultures that rely on symbiotic partners. Thus, it is important to remember that a model for understanding the biology of life on the planet can consist of an individual cultured isolate, mixtures of distinct lineages, or a broad taxonomic group. There is no need to limit studies of models to one of these; ideally, one can study cultures and link those techniques to examine natural populations.

## KEY MICROBES IN EARTH ECOSYSTEMS

From the subsurface to the atmosphere, our understanding of the composition of microbial communities has advanced considerably with the advent of diversity surveys using both DNA sequencing and microscopy. When considering ideal targets for model organisms on our planet, we think it is important to choose Bacteria and Archaea that are ubiquitous, dominant members of the community, and are involved in key biogeochemical processes of that habitat. For example, when we consider the surface oceans, we should focus on groups capable of photosynthesis and carbon cycling.

**Fig 1:**
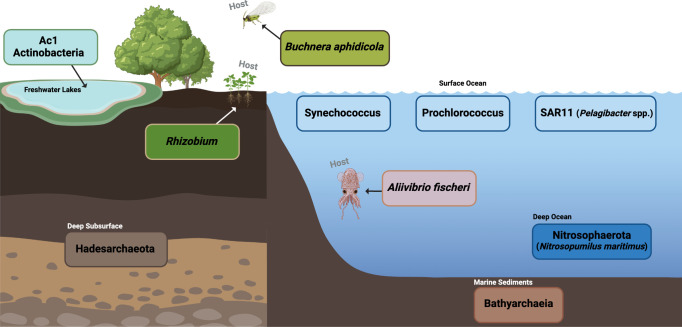
Dominant members of the microbial communities across Earth’s ecosystems. Schematic created with BioRender.com.

Despite localized variations, it has become clear that the surface oceans are dominated by Bacteria, and Archaea dominate the deep oceans (Fig. 1). These organisms have streamlined cell volumes and genomes that are adapted to survive in low-nutrient open oceans. The upper waters consist primarily of a group referred to as “SAR11” (17, 18), cyanobacteria belonging to genera *Prochlorococcus* (19) and *Synechococcus* (20) (Fig. 1). Considering the volume of the oceans, it is easy to see these lineages are among the most dominant on the planet. SAR11 has a global estimated population size of 2.4 × 10^28^ cells and comprises ~25% of the cells in the ocean (21) Table 1. Cultured representatives of all these bacteria exist; however, SAR11, named *Pelagibacter* spp., is not easily cultivated due to their slow growth and other factors including the fact that commonly used plastics for laboratory culturing are toxic to them (22).

**TABLE 1:** Dominant microbes across key ecosystems and cultured representatives proposed here as potential model organisms in the future, given their significant roles in their given ecosystem

Environment	Phyla	Representative	Cultured?	References
Surface ocean	Pseudomonadota	Pelagibacter spp.	Yes	(22)
	Cyanobacteria	*Prochlorococcus*	Yes	(19)
		*Synechococcus*	Yes	(20)
Deep ocean	Nitrosophaerota	*N. maritimus*	Yes	(23)
Marine sediments	Bathyarchaeia	*Bathyarchaeum tardum*	Yes	(24)
Deep subsurface	Hadesarchaeaota	Hadesarchaea	No	(25)
Freshwater lakes	Actinobacteria	Ca. Planktophila	Yes	(26) (27)
Symbionts	Pseudomonadota	*Aliivibrio fischeri*	Yes	(28)
		*Buchnera aphidicola*	No	(29) (30)
		*Rhizobium*	Yes	(31)

Strikingly, archaea constitute the majority (up to 69%) of the cells in the deep sea, with members of the Thaumarchaeota (now named the Nitrosophaerota phylum) dominating with up to 39% of the cells in the deep sea (32, 33). The first member of Thaumarchaeota cultured, *N. maritimus*, confirmed that they are capable of ammonia oxidation (23). Ammonia oxidation is coupled to nitrite oxidation to produce nitrate, a key nutrient for primary productivity. Isotopic analyses have demonstrated that these archaea may be responsible for 30% of the nitrous oxide in the world’s atmosphere (34).

In freshwater lakes, there is also a clear dominant group of Bacteria within the Actinobacteria, group acI (35). Like other bacterioplankton (bacteria that inhabit the water column), the acI are relatively challenging to culture and require high throughput approaches (26, 27). Remarkably, these bacteria are dominant in lakes around the world, and their ability to harness light via actinorhodopsins and organic carbon utilization makes them important players in terms of climate change (36).

Soils and marine sediments are where things become less clear in terms of a single dominant group. These environments tend to be more heterogeneous and certainly considerably more diverse (37). However, there are certain phyla that are ubiquitous such as Bathyarchaeia on the ocean floor and Proteobacteria in soils (38). However, the Proteobacteria is one of the most diverse groups in the Bacteria, and it is not clear that there is one specific lineage that stands out in distribution and abundance. Due to the diversity of soils, more work needs to be done to determine which taxa fit this billing. One could agree this is true in the subsurface as well; however, there are certain taxa that are ubiquitous and often dominate key niches. For example, Bathyarchaeia could be considered an ideal model group for sediments as it is known to be a key player in the recycling of detrital organic matter from the overlying water column (39). Recently, the first pure culture of Bathyarchaeia was obtained from anaerobic lake sediment, *Bathyarchaeum tardum*, which supported dependency on detrital organic matter, specifically oligomers (24).

The deep subsurface is a dynamic and heterogeneous environment that starts a few meters below the surface. One ubiquitous archaeal group that can thrive in a broad range of anoxic subsurface environments (4°C–80°C) is the phylum Hadesarchaeota (25). These archaea are uniquely adapted to utilize substrates generated via subsurface geological processes, carbon monoxide, and hydrocarbons (40). In contrast to the deep subsurface, the atmosphere also contains microbial communities, though these communities are likely far less metabolically active (41). Bacteria found in the atmosphere are likely involved in hydrogen, carbon (specifically methane, carbon dioxide, and carbon monoxide), and nitrogen cycling (42, 43). Recent studies have shown that the dominant microbes present in the atmospheric boundary layer are Alphaproteobacteria and Gammaproteobacteria (42, 43).

## MODEL MICROBES IN HOST-ASSOCIATION INTERACTIONS

Interactions between microbes and eukaryotes, as well as other prokaryotes, have shaped the ecology and evolution of the planet (44). The diversity of host associations in nature makes it challenging to define good model systems to advance our understanding of the symbiotic processes. Furthermore, complicating this multiple microbial species can inhabit hosts, like Wolbachia and insects (45). We now know that huge swaths of microbial diversity (CPR [candidate phyla radiation] and DPANN archaea) consist of small cells that likely require another species to survive (46, 47). However, the host range of most of these lineages has not been determined. There is one member of the Saccharibacteria (formerly TM7) that has been shown to be a ubiquitous human oral episymbiont. A strain belonging to this group has been cultured *Nanosynbacter lyticus*, making interaction experiments more feasible (48, 49). As more of these enigmatic small organisms are studied, our understanding of this vast diversity will reveal new mechanisms of symbiotic interactions.

Some symbiotic bacteria have been extensively studied, and we suggest would be models for different types of interactions. Notably, *Aliivibrio fischeri*, which interacts with squid and fish (28), and *Buchnera*, the obligate endosymbionts of aphids (29, 30). These interactions are fundamentally different, and *Buchnera* is vertically inherited and is mostly beneficial to the host, while *A. fischeri* colonizes the host at each generation. That makes the mechanisms distinct, and both should be models. Perhaps the most well studied of all interactions is between *Rhizobium* and plant roots, which makes this another potential system on which decades of studies can be founded (31).

## UNDERSTANDING KEY TRANSITION IN THE ORIGINS OF COMPLEX LIFE

Not only should prominence in nature be a metric for a model organism but also significance in the evolution of life on the planet. For example, the origins of organelles like mitochondria from Alphaproteobacteria (50) and chloroplasts from cyanobacteria via endosymbiosis. Certainly, one of the major transitions of life was the origin of complex cellular organization in eukaryotes. The past decade has revealed that a group of Archaea named Asgards (now phylum Asgardarchaeota) were pivotal in this event (51). The first to be described was Lokiarchaeia, then soon after several others were identified, including Odinarchaeia, Thorarchaeia, and Heimdallarchaeia (52). Two cultures of Lokiarchaeia representatives from this clade have been obtained (53, 54); however, these are difficult to maintain as well. Not only are these Archaea related to eukaryotes on the tree of life, but they also have a wide variety of proteins that had previously been thought to be exclusive to eukaryotes. These building blocks of complex life from uncultured Asgards have been heterologically expressed, and their functions have been determined (55–57). These novel proteins from these uncultured lineages have been functionally characterized at the structural level using crystallization (58).

## CULTURE-INDEPENDENT TECHNIQUES TO UNDERSTAND MODEL ENVIRONMENTAL ORGANISMS

Classically, model organisms are easy to maintain in cultures, readily manipulated (via genetics), cryopreserved, and broadly available to everyone. This is not the case for many of the organisms discussed above and other dominant lineages. While we now have cultured representatives of them, they are difficult to grow in the lab, and they all lack established genetic systems. Regardless, having cultures of these globally important organisms is extremely valuable. Much has been learned from cultures of *Prometheoarchaeum syntrophicum* (Lokiarchaeia), *Nitrosopulmilus* spp. (Thaumarchaeota), and *Pelagibacter* spp. (SAR11—Proteobacteria). For example, high-resolution images have been generated of the *N. maritimus* surface layer (S-layer) using electron cryotomography and single-particle cryomicroscopy revealing that they can sieve/concentrate ammonium in oligotrophic waters (59). This combined with these archaea’s remarkable versatility to utilize a variety of nitrogen substrates is what makes them predominant players in ocean nutrient cycling (60, 61). This reminds us that we must always have an environmental perspective of what we discover. Studies on physiology, biochemistry, regulation, signaling, interactions, pathogenesis, evolution, and other aspects of an organism in isolation in a laboratory are going to differ from nature.

The distinction between culture-based and culture-independent is not black and white. It is common to run experiments using inoculum from the environment to study mixed communities, e.g., mesocosms. All the approaches discussed in this section used to study microbes *in situ* can be used to examine mesocosms. One common technique is to add isotopically labeled substrates to track the uptake and flow of carbon and nutrients by extracting the heavy DNA, referred to as DNA-SIP (stable isotope probing). For example, this has been used to resolve the community structure and metabolic pathways involved in organic carbon cycling in soils (62) and oil degradation in the Gulf of Mexico (37).

The most common approach to studying uncultivated microorganisms is metagenomics. Despite the large number of discoveries that have resulted from the reconstruction of metagenomic-assembled genomes (MAGs), there is often skepticism regarding the accuracy of these as true representations of a genome from a cell in nature. This issue is largely due to their reconstruction from mixed communities. They can contain assembly errors, and reads from distinct strains can be mixed into an individual MAG assembly, unless they are carefully curated (63, 64). Also, metagenomic binning can lead to contigs from different species being incorporated into MAGs, usually shorter fragments <20 kb in length. This is not a new concern, and similar discussions occurred with the sequencing of individual 16S rRNA genes decades ago (65). The analyses of MAGs in environmental microbiology are now the norm. Despite this, there has not been a major misrepresentation of communities using this approach. One reason for this is that we rarely obtain 1–2 closely related MAGs; instead, we are analyzing dozens that have a similar genetic composition. This results in a statistically reliable set of genes within a clade.

It is much more powerful to have data from the community rather than a single species that is often not a dominant member of that community. For example, we do not see the presence of fine-scale genetic heterogeneity in MAGs as being an issue. If handled correctly, this can make it possible to track strain-level shifts in populations over time (64). Similar to what was done in the long-term evolution experiment, but in nature (66). Moreover, metagenomics circumvents the reliance on using cultivation to infer the biology of microbes in nature. The microbiology community acknowledges metagenomics as a powerful resource to study uncultivated organisms. This is particularly true given the large amount of data generated, decreasing costs, wide accessibility, and numerous experimental validations of predictions generated through metagenomic analyses (67–70). Understanding the existing limitations of culture-independent approaches are important and should not drive us away from them but allow us to utilize them more effectively and responsibly.

In the past, we have lacked techniques to understand cell biology and activity *in situ* or even know what organisms are present in nature. The advances in metagenomics, transcriptomics, proteomics, microscopy, and geochemistry techniques have enabled us to examine individual cells to entire communities independent of culturing. We can now obtain complete genomes (71), track metabolic activity (72, 73), image biomolecules (46, 74), express genes, confirm function (57), and examine physiologies of uncultured cells (37).

There is an ever-growing toolbox for studying microbes in nature. One powerful example is the use of Raman spectroscopy which measures the inelastic scattering of light to provide detailed information about the molecular composition and metabolic activities of individual cells. This technique enables researchers to analyze microbial cells directly in their natural environments, identifying specific biomolecules, tracking metabolic changes, and monitoring interactions within communities (75). This technique can be combined with other culture-independent approaches to enhance our understanding of the microbial diversity, ecological roles, and functional dynamics of uncultivated microorganisms across various environments. These complementary technologies include nano-scale secondary ion mass spectrometry (nanoSIMS), fluorescence *in situ* hybridization (FISH), cell sorting, and genomic sequencing.

The combination of Raman spectroscopy and nanoSIMS allows us to map the distribution of specific elements within individual cells and semi-quantify microbial metabolic activities (76). When combined with FISH, Raman can identify target microbes by linking spectroscopy with taxonomic information (77). Raman-activated cell sorting enables the collection of target microbes from environmental samples based on specific biological activity of interest (78).

## SUMMARY

Advancements in technology now allow us to study microbes beyond pure cultures. Culture-independent techniques play a crucial role in achieving this goal. When combined, these techniques provide valuable information about the metabolism and physicochemical conditions necessary for the survival of uncultivated organisms. Thus, we hope that there can be a shift from studying classic organisms in culture to more studies looking at the ecologically dominant microbes in the natural habitats to understand them at a molecular level and their impact on the ecosystems.

## References

[1] Leonelli S. 2013. Model organism, p 1398–1401. In Dubitzky W, Wolkenhauer O, Cho KH, Yokota H (ed), Encyclopedia of systems biology. Springer, New York, NY.

[2] Escherich T. 1988. The intestinal bacteria of the neonate and breast-fed infant. 1884. Rev Infect Dis 10:1220–1225. doi:10.1093/clinids/10.6.1220 3060950

[3] Lehman IR, Bessman MJ, Simms ES, Kornberg A. 1958. Enzymatic synthesis of deoxyribonucleic acid. I. Preparation of substrates and partial purification of an enzyme from Escherichia coli. J Biol Chem 233:163–170. 13563462

[4] Luria SE, Delbrück M. 1943. Mutations of bacteria from virus sensitivity to virus resistance. Genetics 28:491–511. doi:10.1093/genetics/28.6.491 17247100 PMC1209226

[5] Cohen SN, Chang ACY, Boyer HW, Helling RB. 1973. Construction of biologically functional bacterial plasmids in vitro*.* Proc Natl Acad Sci U S A 70:3240–3244. doi:10.1073/pnas.70.11.3240 4594039 PMC427208

[6] Rousset F, Cabezas-Caballero J, Piastra-Facon F, Fernández-Rodríguez J, Clermont O, Denamur E, Rocha EPC, Bikard D. 2021. The impact of genetic diversity on gene essentiality within the Escherichia coli species. Nat Microbiol 6:301–312. doi:10.1038/s41564-020-00839-y 33462433

[7] Barák I. 2021. Special Issue “Bacillus subtilis as a model organism to study basic cell processes.” Microorganisms 9:2459. doi:10.3390/microorganisms9122459 34946061 PMC8708606

[8] McLoon AL, Guttenplan SB, Kearns DB, Kolter R, Losick R. 2011. Tracing the domestication of a biofilm-forming bacterium. J Bacteriol 193:2027–2034. doi:10.1128/JB.01542-10 21278284 PMC3133032

[9] Woese CR, Fox GE. 1977. Phylogenetic structure of the prokaryotic domain: the primary kingdoms. Proc Natl Acad Sci U S A 74:5088–5090. doi:10.1073/pnas.74.11.5088 270744 PMC432104

[10] Woese CR, Kandler O, Wheelis ML. 1990. Towards a natural system of organisms: proposal for the domains Archaea, bacteria, and Eucarya. Proc Natl Acad Sci U S A 87:4576–4579. doi:10.1073/pnas.87.12.4576 2112744 PMC54159

[11] Wolfe RS. 2006. The Archaea: a personal overview of the formative years, p 3–9. In Dworkin M, Falkow S, Rosenberg E, Schleifer KH, Stackebrandt E (ed), The prokaryotes: Archaea. Bacteria: firmicutes, Actinomycetes. Vol. 3. Springer, New York, NY.

[12] Brock TD, Brock KM, Belly RT, Weiss RL. 1972. Sulfolobus: a new genus of sulfur-oxidizing bacteria living at low pH and high temperature. Archiv Mikrobiol 84:54–68. doi:10.1007/BF00408082 4559703

[13] Oesterhelt D, Stoeckenius W. 1971. Rhodopsin-like protein from the purple membrane of Halobacterium halobium. Nat New Biol 233:149–152. doi:10.1038/newbio233149a0 4940442

[14] Chen L, Brügger K, Skovgaard M, Redder P, She Q, Torarinsson E, Greve B, Awayez M, Zibat A, Klenk H-P, Garrett RA. 2005. The genome of Sulfolobus acidocaldarius, a model organism of the Crenarchaeota. J Bacteriol 187:4992–4999. doi:10.1128/JB.187.14.4992-4999.2005 15995215 PMC1169522

[15] Leigh JA, Albers S-V, Atomi H, Allers T. 2011. Model organisms for genetics in the domain Archaea: methanogens, halophiles, Thermococcales and Sulfolobales. FEMS Microbiol Rev 35:577–608. doi:10.1111/j.1574-6976.2011.00265.x 21265868

[16] Prangishvili D, Albers SV, Holz I, Arnold HP, Stedman K, Klein T, Singh H, Hiort J, Schweier A, Kristjansson JK, Zillig W. 1998. Conjugation in archaea: frequent occurrence of conjugative plasmids in Sulfolobus. Plasmid 40:190–202. doi:10.1006/plas.1998.1363 9806856

[17] Giovannoni SJ, Britschgi TB, Moyer CL, Field KG. 1990. Genetic diversity in Sargasso Sea bacterioplankton. Nature New Biol 345:60–63. doi:10.1038/345060a0 2330053

[18] Morris RM, Rappé MS, Connon SA, Vergin KL, Siebold WA, Carlson CA, Giovannoni SJ. 2002. SAR11 clade dominates ocean surface bacterioplankton communities. Nature New Biol 420:806–810. doi:10.1038/nature01240 12490947

[19] Chisholm SW, Olson RJ, Zettler ER, Goericke R, Waterbury JB, Welschmeyer NA. 1988. A novel free-living prochlorophyte abundant in the oceanic euphotic zone. Nature New Biol 334:340–343. doi:10.1038/334340a0

[20] Waterbury JB, Watson SW, Guillard RRL, Brand LE. 1979. Widespread occurrence of a unicellular, marine, planktonic, cyanobacterium. Nature New Biol 277:293–294. doi:10.1038/277293a0

[21] Giovannoni SJ. 2017. SAR11 bacteria: the most abundant plankton in the oceans. Annu Rev Mar Sci 9:231–255. doi:10.1146/annurev-marine-010814-015934 27687974

[22] Rappé MS, Connon SA, Vergin KL, Giovannoni SJ. 2002. Cultivation of the ubiquitous SAR11 marine bacterioplankton clade. Nature New Biol 418:630–633. doi:10.1038/nature00917 12167859

[23] Könneke M, Bernhard AE, de la Torre JR, Walker CB, Waterbury JB, Stahl DA. 2005. Isolation of an autotrophic ammonia-oxidizing marine archaeon. Nature New Biol 437:543–546. doi:10.1038/nature03911 16177789

[24] Khomyakova MA, Merkel AY, Mamiy DD, Klyukina AA, Slobodkin AI. 2023. Phenotypic and genomic characterization of Bathyarchaeum tardum gen. nov., sp. nov., a cultivated representative of the archaeal class Bathyarchaeia. Front Microbiol 14:1214631. doi:10.3389/fmicb.2023.1214631 37675420 PMC10477458

[25] Baker BJ, Saw JH, Lind AE, Lazar CS, Hinrichs K-U, Teske AP, Ettema TJG. 2016. Genomic inference of the metabolism of cosmopolitan subsurface Archaea, Hadesarchaea. Nat Microbiol 1:16002. doi:10.1038/nmicrobiol.2016.2 27572167

[26] Kang I, Kim S, Islam MR, Cho J-C. 2017. The first complete genome sequences of the acI lineage, the most abundant freshwater Actinobacteria, obtained by whole-genome-amplification of dilution-to-extinction cultures. Sci Rep 7:42252. doi:10.1038/srep42252 28186143 PMC5301498

[27] Kim S, Kang I, Seo J-H, Cho J-C. 2019. Culturing the ubiquitous freshwater actinobacterial acI lineage by supplying a biochemical “helper” catalase. ISME J 13:2252–2263. doi:10.1038/s41396-019-0432-x 31073214 PMC6775976

[28] Septer AN. 2019. TheVibrio-squid symbiosis as a model for studying interbacterial competition. mSystems 4. doi:10.1128/mSystems.00108-19 PMC6584871 31186308

[29] Chong RA, Park H, Moran NA. 2019. Genome evolution of the obligate endosymbiont Buchnera aphidicola. Mol Biol Evol 36:1481–1489. doi:10.1093/molbev/msz082 30989224

[30] Moran NA. 2021. Microbe profile: Buchnera aphidicola: ancient aphid accomplice and endosymbiont exemplar. Microbiology (Reading) 167:001127. doi:10.1099/mic.0.001127 34939561 PMC10228527

[31] van Rhijn P, Vanderleyden J. 1995. The rhizobium-plant symbiosis. Microbiol Rev 59:124–142. doi:10.1128/mr.59.1.124-142.1995 7708010 PMC239357

[32] Karner MB, DeLong EF, Karl DM. 2001. Archaeal dominance in the mesopelagic zone of the Pacific Ocean. Nature New Biol 409:507–510. doi:10.1038/35054051 11206545

[33] Fuhrman JA, McCallum K, Davis AA. 1992. Novel major archaebacterial group from marine plankton. Nature New Biol 356:148–149. doi:10.1038/356148a0 1545865

[34] Santoro AE, Buchwald C, McIlvin MR, Casciotti KL. 2011. Isotopic signature of N(2)O produced by marine ammonia-oxidizing archaea. Science 333:1282–1285. doi:10.1126/science.1208239 21798895

[35] Newton RJ, Jones SE, Eiler A, McMahon KD, Bertilsson S. 2011. A guide to the natural history of freshwater lake bacteria. Microbiol Mol Biol Rev 75:14–49. doi:10.1128/MMBR.00028-10 21372319 PMC3063352

[36] Dwulit-Smith JR, Hamilton JJ, Stevenson DM, He S, Oyserman BO, Moya-Flores F, Garcia SL, Amador-Noguez D, McMahon KD, Forest KT. 2018. acI actinobacteria assemble a functional actinorhodopsin with natively synthesized retinal. Appl Environ Microbiol 84:e01678–18. doi:10.1128/AEM.01678-18 30315080 PMC6275354

[37] Dombrowski N, Donaho JA, Gutierrez T, Seitz KW, Teske AP, Baker BJ. 2016. Reconstructing metabolic pathways of hydrocarbon-degrading bacteria from the deepwater horizon oil spill. Nat Microbiol 1:16057. doi:10.1038/nmicrobiol.2016.57 27572965

[38] Janssen PH. 2006. Identifying the dominant soil bacterial taxa in libraries of 16S rRNA and 16S rRNA genes. Appl Environ Microbiol 72:1719–1728. doi:10.1128/AEM.72.3.1719-1728.2006 16517615 PMC1393246

[39] Lloyd KG, Schreiber L, Petersen DG, Kjeldsen KU, Lever MA, Steen AD, Stepanauskas R, Richter M, Kleindienst S, Lenk S, Schramm A, Jørgensen BB. 2013. Predominant archaea in marine sediments degrade detrital proteins. Nature New Biol 496:215–218. doi:10.1038/nature12033 23535597

[40] Wang Y, Wegener G, Hou J, Wang F, Xiao X. 2019. Expanding anaerobic alkane metabolism in the domain of Archaea. Nat Microbiol 4:595–602. doi:10.1038/s41564-019-0364-2 30833728

[41] Burrows SM, Butler T, Jöckel P, Tost H, Kerkweg A, Pöschl U, Lawrence MG. 2009. Bacteria in the global atmosphere – part 2: modeling of emissions and transport between different ecosystems. Atmos Chem Phys 9:9281–9297. doi:10.5194/acp-9-9281-2009

[42] Šantl-Temkiv T, Amato P, Casamayor EO, Lee PKH, Pointing SB. 2022. Microbial ecology of the atmosphere. FEMS Microbiol Rev 46:fuac009. doi:10.1093/femsre/fuac009 35137064 PMC9249623

[43] Archer S, Lee K, Caruso T, Leung M, Tong X, Salter SJ, Hinchliffe G, Maki T, Santl-Temkiv T, Warren-Rhodes K, et al.. 2022. Global biogeography of atmospheric microorganisms reflects diverse recruitment and environmental filtering. In Review. doi:10.21203/rs.3.rs-244923/v4

[44] Perreau J, Moran NA. 2022. Genetic innovations in animal-microbe symbioses. Nat Rev Genet 23:23–39. doi:10.1038/s41576-021-00395-z 34389828 PMC8832400

[45] Misof B, Liu S, Meusemann K, Peters RS, Donath A, Mayer C, Frandsen PB, Ware J, Flouri T, Beutel RG, et al.. 2014. Phylogenomics resolves the timing and pattern of insect evolution. Science 346:763–767. doi:10.1126/science.1257570 25378627

[46] Baker BJ, Comolli LR, Dick GJ, Hauser LJ, Hyatt D, Dill BD, Land ML, Verberkmoes NC, Hettich RL, Banfield JF. 2010. Enigmatic, ultrasmall, uncultivated Archaea. Proc Natl Acad Sci U S A 107:8806–8811. doi:10.1073/pnas.0914470107 20421484 PMC2889320

[47] Hug LA, Baker BJ, Anantharaman K, Brown CT, Probst AJ, Castelle CJ, Butterfield CN, Hernsdorf AW, Amano Y, Ise K, Suzuki Y, Dudek N, Relman DA, Finstad KM, Amundson R, Thomas BC, Banfield JF. 2016. A new view of the tree of life. Nat Microbiol 1:16048. doi:10.1038/nmicrobiol.2016.48 27572647

[48] He X, McLean JS, Edlund A, Yooseph S, Hall AP, Liu S-Y, Dorrestein PC, Esquenazi E, Hunter RC, Cheng G, Nelson KE, Lux R, Shi W. 2015. Cultivation of a human-associated TM7 phylotype reveals a reduced genome and epibiotic parasitic lifestyle. Proc Natl Acad Sci USA 112:244–249. doi:10.1073/pnas.1419038112 25535390 PMC4291631

[49] Zhong Q, Liao B, Liu J, Shen W, Wang J, Wei L, Ma Y, Dong P-T, Bor B, McLean JS, Chang Y, Shi W, Cen L, Wu M, Liu J, Li Y, He X, Le S. 2024. Episymbiotic Saccharibacteria TM7x modulates the susceptibility of its host bacteria to phage infection and promotes their coexistence. Proc Natl Acad Sci USA 121. doi:10.1073/pnas.2319790121 PMC11032452 38593079

[50] Martijn J, Vosseberg J, Guy L, Offre P, Ettema TJG. 2018. Deep mitochondrial origin outside the sampled alphaproteobacteria. Nature New Biol 557:101–105. doi:10.1038/s41586-018-0059-5 29695865

[51] Tamarit D, Köstlbacher S, Appler KE, Panagiotou K, De Anda V, Rinke C, Baker BJ, Ettema TJG. 2024. Description of Asgardarchaeum abyssi gen. nov. spec. nov., a novel species within the class Asgardarchaeia and phylum Asgardarchaeota in accordance with the SeqCode. Syst Appl Microbiol 47:126525. doi:10.1016/j.syapm.2024.126525 38909391

[52] Zaremba-Niedzwiedzka K, Caceres EF, Saw JH, Bäckström D, Juzokaite L, Vancaester E, Seitz KW, Anantharaman K, Starnawski P, Kjeldsen KU, Stott MB, Nunoura T, Banfield JF, Schramm A, Baker BJ, Spang A, Ettema TJG. 2017. Asgard archaea illuminate the origin of eukaryotic cellular complexity. Nat. New Biol. 541:353–358. doi:10.1038/nature21031 28077874

[53] Imachi H, Nobu MK, Nakahara N, Morono Y, Ogawara M, Takaki Y, Takano Y, Uematsu K, Ikuta T, Ito M, Matsui Y, Miyazaki M, Murata K, Saito Y, Sakai S, Song C, Tasumi E, Yamanaka Y, Yamaguchi T, Kamagata Y, Tamaki H, Takai K. 2020. Isolation of an archaeon at the prokaryote-eukaryote interface. Nat New Biol 577:519–525. doi:10.1038/s41586-019-1916-6 PMC7015854 31942073

[54] Rodrigues-Oliveira T, Wollweber F, Ponce-Toledo RI, Xu J, Rittmann S-M, Klingl A, Pilhofer M, Schleper C. 2023. Actin cytoskeleton and complex cell architecture in an asgard archaeon. Nat New Biol 613:332–339. doi:10.1038/s41586-022-05550-y PMC9834061 36544020

[55] Akıl C, Robinson RC. 2018. Genomes of asgard archaea encode profilins that regulate actin. Nat New Biol 562:439–443. doi:10.1038/s41586-018-0548-6 30283132

[56] Shomar H, Georjon H, Feng Y, Olympio B, Tesson F, Cury J, Wu F, Bernheim A. 2023. Viperin immunity evolved across the tree of life through serial innovations on a conserved scaffold. Nat Ecol Evol. doi:10.1038/s41559-024-02463-z 38965412

[57] Leao P, Little ME, Appler KE, Sahaya D, Aguilar-Pine E, Currie K, Finkelstein IJ, De Anda V, Baker BJ. 2023. Asgard archaea defense systems and their roles in the origin of eukaryotic immunity. Nature Communications. doi:10.1038/s41467-024-50195-2 PMC11291487 39085212

[58] Tran LT, Akıl C, Senju Y, Robinson RC. 2024. The eukaryotic-like characteristics of small GTPase, roadblock and TRAPPC3 proteins from asgard archaea. Commun Biol 7:273. doi:10.1038/s42003-024-05888-1 38472392 PMC10933270

[59] von Kügelgen A, Cassidy CK, van Dorst S, Pagani LL, Batters C, Ford Z, Löwe J, Alva V, Stansfeld PJ, Bharat TAM. 2024. Membraneless channels sieve cations in ammonia-oxidizing marine archaea. Nat. New Biol. 630:230–236. doi:10.1038/s41586-024-07462-5 PMC11153153 38811725

[60] Kitzinger K, Padilla CC, Marchant HK, Hach PF, Herbold CW, Kidane AT, Könneke M, Littmann S, Mooshammer M, Niggemann J, Petrov S, Richter A, Stewart FJ, Wagner M, Kuypers MMM, Bristow LA. 2019. Cyanate and urea are substrates for nitrification by Thaumarchaeota in the marine environment. Nat Microbiol 4:234–243. doi:10.1038/s41564-018-0316-2 30531977 PMC6825518

[61] Palatinszky M, Herbold C, Jehmlich N, Pogoda M, Han P, von Bergen M, Lagkouvardos I, Karst SM, Galushko A, Koch H, Berry D, Daims H, Wagner M. 2015. Cyanate as an energy source for nitrifiers. Nature New Biol 524:105–108. doi:10.1038/nature14856 PMC4539577 26222031

[62] Starr EP, Shi S, Blazewicz SJ, Koch BJ, Probst AJ, Hungate BA, Pett-Ridge J, Firestone MK, Banfield JF. 2021. Stable-isotope-informed, genome-resolved metagenomics uncovers potential cross-kingdom interactions in rhizosphere soil. mSphere 6:e0008521. doi:10.1128/mSphere.00085-21 34468166 PMC8550312

[63] Denef VJ, Kalnejais LH, Mueller RS, Wilmes P, Baker BJ, Thomas BC, VerBerkmoes NC, Hettich RL, Banfield JF. 2010. Proteogenomic basis for ecological divergence of closely related bacteria in natural acidophilic microbial communities. Proc Natl Acad Sci U S A 107:2383–2390. doi:10.1073/pnas.0907041107 20133593 PMC2823883

[64] Rohwer RR, Kirkpatrick M, Garcia SL, Kellom M, McMahon KD, Baker BJ. 2024. Bacterial ecology and evolution converge on seasonal and decadal scales. bioRxiv:2024.02.06.579087. doi:10.1101/2024.02.06.579087

[65] Hugenholtz P, Huber T. 2003. Chimeric 16S rDNA sequences of diverse origin are accumulating in the public databases. Int J Syst Evol Microbiol 53:289–293. doi:10.1099/ijs.0.02441-0 12656186

[66] Lenski RE. 2017. Experimental evolution and the dynamics of adaptation and genome evolution in microbial populations. ISME J 11:2181–2194. doi:10.1038/ismej.2017.69 28509909 PMC5607360

[67] Ettwig KF, Butler MK, Le Paslier D, Pelletier E, Mangenot S, Kuypers MMM, Schreiber F, Dutilh BE, Zedelius J, de Beer D, Gloerich J, Wessels HJCT, van Alen T, Luesken F, Wu ML, van de Pas-Schoonen KT, Op den Camp HJM, Janssen-Megens EM, Francoijs K-J, Stunnenberg H, Weissenbach J, Jetten MSM, Strous M. 2010. Nitrite-driven anaerobic methane oxidation by oxygenic bacteria. Nature New Biol 464:543–548. doi:10.1038/nature08883 20336137

[68] Borrel G, Adam PS, McKay LJ, Chen L-X, Sierra-García IN, Sieber CMK, Letourneur Q, Ghozlane A, Andersen GL, Li W-J, Hallam SJ, Muyzer G, de Oliveira VM, Inskeep WP, Banfield JF, Gribaldo S. 2019. Wide diversity of methane and short-chain alkane metabolisms in uncultured archaea. Nat Microbiol 4:603–613. doi:10.1038/s41564-019-0363-3 30833729 PMC6453112

[69] Zhou Z, Zhang C-J, Liu P-F, Fu L, Laso-Pérez R, Yang L, Bai L-P, Li J, Yang M, Lin J-Z, Wang W-D, Wegener G, Li M, Cheng L. 2022. Non-syntrophic methanogenic hydrocarbon degradation by an archaeal species. Nature New Biol 601:257–262. doi:10.1038/s41586-021-04235-2 34937940

[70] Ostrowski MP, La Rosa SL, Kunath BJ, Robertson A, Pereira G, Hagen LH, Varghese NJ, Qiu L, Yao T, Flint G, et al.. 2022. Mechanistic insights into consumption of the food additive xanthan gum by the human gut microbiota. Nat Microbiol 7:556–569. doi:10.1038/s41564-022-01093-0 35365790 PMC11537241

[71] Chen L-X, Anantharaman K, Shaiber A, Eren AM, Banfield JF. 2020. Accurate and complete genomes from metagenomes. Genome Res 30:315–333. doi:10.1101/gr.258640.119 32188701 PMC7111523

[72] Li M, Baker BJ, Anantharaman K, Jain S, Breier JA, Dick GJ. 2015. Genomic and transcriptomic evidence for scavenging of diverse organic compounds by widespread deep-sea archaea. Nat Commun 6:8933. doi:10.1038/ncomms9933 26573375 PMC4660358

[73] Ram RJ, Verberkmoes NC, Thelen MP, Tyson GW, Baker BJ, Blake RC, Shah M, Hettich RL, Banfield JF. 2005. Community proteomics of a natural microbial biofilm. Science 308:1915–1920. 15879173

[74] Comolli LR, Banfield JF. 2014. Inter-species interconnections in acid mine drainage microbial communities. Front Microbiol 5:367. doi:10.3389/fmicb.2014.00367 25120533 PMC4110969

[75] Rebrosova K, Samek O, Kizovsky M, Bernatova S, Hola V, Ruzicka F. 2022. Raman spectroscopy-A novel Method for identification and characterization of microbes on a single-cell level in clinical settings. Front Cell Infect Microbiol 12:866463. doi:10.3389/fcimb.2022.866463 35531343 PMC9072635

[76] Schaible GA, Kohtz AJ, Cliff J, Hatzenpichler R. 2022. Correlative SIP-FISH-Raman-SEM-NanoSIMS links identity, morphology, biochemistry, and physiology of environmental microbes. ISME Commun 2:52. doi:10.1038/s43705-022-00134-3 37938730 PMC9723565

[77] Huang WE, Stoecker K, Griffiths R, Newbold L, Daims H, Whiteley AS, Wagner M. 2007. Raman‐FISH: combining stable‐isotope raman spectroscopy and fluorescencein situ hybridization for the single cell analysis of identity and function. Environ Microbiol 9:1878–1889. doi:10.1111/j.1462-2920.2007.01352.x 17635536

[78] Li J, Zhang D, Luo C, Li B, Zhang G. 2023. In situ discrimination and cultivation of active degraders in soils by genome-directed cultivation assisted by SIP-raman-activated cell sorting. Environ Sci Technol 57:17087–17098. doi:10.1021/acs.est.3c04247 37823365

